# Knockdown of *Son*, a mouse homologue of the ZTTK syndrome gene, causes neuronal migration defects and dendritic spine abnormalities

**DOI:** 10.1186/s13041-020-00622-4

**Published:** 2020-05-24

**Authors:** Masashi Ueda, Tohru Matsuki, Masahide Fukada, Shima Eda, Akie Toya, Akio Iio, Hidenori Tabata, Atsuo Nakayama

**Affiliations:** 1 Department of Cellular Pathology, Institute for Developmental Research, Aichi Developmental Disability Center, Kasugai, Aichi 4800392 Japan; 2 Department of Neurobiochemistry, Nagoya University School of Medicine, Nagoya, Aichi 4668560 Japan; 3 Biogate Co. Ltd., 331-1 Ohmori, Yamagata, Gifu, 5012123 Japan; 4 Department of Molecular Neurobiology, Institute for Developmental Research, Aichi Developmental Disability Center, Kasugai, Aichi 4800392 Japan

**Keywords:** SON, Zhu-Tokita-Takenouchi-Kim syndrome, Brain malformation, Intellectual disability, Neuronal migration, Spinogenesis

## Abstract

Zhu-Tokita-Takenouchi-Kim (ZTTK) syndrome, a rare congenital anomaly syndrome characterized by intellectual disability, brain malformation, facial dysmorphism, musculoskeletal abnormalities, and some visceral malformations is caused by de novo heterozygous mutations of the *SON* gene. The nuclear protein SON is involved in gene transcription and RNA splicing; however, the roles of SON in neural development remain undetermined. We investigated the effects of *Son* knockdown on neural development in mice and found that *Son* knockdown in neural progenitors resulted in defective migration during corticogenesis and reduced spine density on mature cortical neurons. The induction of human wild-type SON expression rescued these neural abnormalities, confirming that the abnormalities were caused by SON insufficiency. We also applied truncated SON proteins encoded by disease-associated mutant *SON* genes for rescue experiments and found that a truncated SON protein encoded by the most prevalent *SON* mutant found in ZTTK syndrome rescued the neural abnormalities while another much shorter mutant SON protein did not. These data indicate that SON insufficiency causes neuronal migration defects and dendritic spine abnormalities, which seem neuropathological bases of the neural symptoms of ZTTK syndrome. In addition, the results support that the neural abnormalities in ZTTK syndrome are caused by SON haploinsufficiency independent of the types of mutation that results in functional or dysfunctional proteins.

## Introduction

Recent genetic studies identified 31 individuals exhibiting intellectual disability (ID) and/or developmental delay with de novo heterozygous mutations in *SON*, which was established as Zhu-Tokita-Takenouchi-Kim (ZTTK) syndrome [1–6]. ZTTK syndrome was further characterized as a congenital anomaly syndrome of ID, brain malformation, facial dysmorphism, musculoskeletal abnormalities, and less common visceral malformations [1, 2, 4]. The mutations found to be associated with ZTTK syndrome are mostly frameshift mutations and nonsense substitutions generating a premature termination codon [1–6], and transcripts of the mutant gene seem to be degraded due to nonsense-mediated mRNA decay (NMD) [1]; this has made ZTTK syndrome to be regarded as an entity caused by *SON* haploinsufficiency.

*SON* is a ubiquitously expressed and evolutionarily conserved gene in vertebrates and is located on the human chromosome region 21q22.11 [4]. It encodes the DNA- and RNA-binding protein SON, which functions in RNA splicing as well as gene repression [7–13]. A wide variety of genes are, thus, under the control of SON, and SON has been reported to be involved in cell cycle regulation and stem cell maintenance [7–12]. However, the functional significance of SON in neural development is largely unknown, and the pathological consequence of *SON* haploinsufficiency underlying the neural phenotypes of ZTTK syndrome, such as ID and brain malformation, remains undetermined. In this report, we revealed through knockdown experiments in the developing mouse brain that *Son* insufficiency caused neuronal migration abnormalities and reduced spine density. Rescue experiments that induced the expression of human wild-type SON protein and truncated SON proteins encoded by disease-associated mutant *SON* genes provided further information relevant to the pathophysiology of ZTTK syndrome.

## Materials and methods

### Animals

All animals were used in accordance with an animal protocol approved by the Animal Care and Use Committee of the Institute for Developmental Research, Aichi Developmental Disability Center. Timed-pregnant ICR mice were purchased from Japan SLC (Hamamatsu, Japan).

### Antibodies

We raised an antibody against mouse SON by immunizing rabbits with a keyhole limpet hemocyanin-fused mouse SON peptide (NM_178880, residues 4–23) (Biomatik, Wilmington, DE). The other antibodies used in this study were as follows: anti-HA (6E2, 2367) (Cell Signaling Technology, Beverly, MA), anti-β-actin (AC-15, A5441), anti-SRSF2/SC35 (SC-35, SAB4200725) (Sigma-Aldrich, St. Louis, MO), anti-green fluorescent protein (GFP) (A10262), Alexa-conjugated secondary antibodies (Invitrogen, Waltham, MA), and horseradish peroxidase (HRP)-conjugated secondary antibodies (Jackson ImmunoResearch, West Grove, PA).

### Plasmid construction

Short hairpin RNA (shRNA) vectors were constructed by inserting cDNAs of the following shRNAs into the pLLC vector [14]: shRNA#1 (5′-AGGCTCAATTACTTGAAATA-3′) [8] and shRNA#2 (5′-GCTGAGCGTTCTATGATGT-3′) [15]. The pCAGGS vector, kindly provided by Dr. Miyazaki in Osaka University, was engineered to express HA-tagged human SON (hSON), shRNA-resistant human SON (hSONr), or a disease-associated mutant SON (hSONm1 or hSONm2). hSONr was derived from *SON* cDNA containing three nucleotide substitutions within the target sequence of shRNA#1.

### Cell culture

Neuro-2a and HEK293 cells were obtained from ATCC (Manassas, VA) and RIKEN BRC (Tsukuba, Japan), respectively. Each cell line was maintained with DMEM supplemented with 10% FBS, penicillin, and streptomycin under standard conditions. The expression plasmids were transfected with polyethyleneimine (Polysciences, Inc., Warrington, PA) or Lipofectamine 2000 (Invitrogen), according to manufacturer’s directions.

### In utero electroporation (IUE)

Various combinations of plasmids were transfected into neural progenitors on the lateral ventricular surface of E14.5 embryos by IUE as previously described [16]. Electroporation was performed by administering five consequent electronic pulses at an intensity of 35 V for a duration of 50 ms with 450-ms intervals using a NEPA21 SuperElectroporator (NEPA Gene, Chiba, Japan). For neuronal migration analysis, 1 μg of shRNA vector with 1.5 μg of the pCAGGS vectors harboring the various forms of human *SON* cDNA described above were applied. For dendritic spine formation analysis, a plasmid mixture containing 1 μg of shRNA vector with or without 0.5 μg of the hSON expression vectors was applied.

### Immunocytochemistry and immunohistochemistry

Neuro-2a cells were grown on poly-L-lysine coated glass coverslips. The cells were fixed with 4% paraformaldehyde and permeabilized with 0.1% Triton X-100. Immunocytochemical staining was performed with the anti-SON antibody and Alexa-conjugated secondary antibodies (Invitrogen).

Paraffin-embedded brain tissues were sectioned at a thickness of 4 μm and subjected to immunohistochemical staining and hematoxylin and eosin (HE) staining. Anti-SON immunoreactivity was visualized using EnVision (Dako, Glostrup, Denmark). All images were acquired with BX60 microscope (Olympus, Tokyo, Japan).

### Neuronal migration analysis and spine density analysis

Mice subjected to IUE were sacrificed at E18.5 or P60, and perfused with 4% paraformaldehyde in PBS. Then the mice were dissected and obtained brains were postfixed in paraformaldehyde solution for 2–24 h. The brains were embedded in 3% agarose and sectioned at a thickness of 50–100 μm using a VT1200S vibrating microtome (Leica Microsystems Wetzlar, Germany). The coronal sections were stained with an anti-GFP antibody to visualize *Son* knockdown cells. The distribution of GFP-positive cells at E18.5 was examined to assess neuronal migration. For spine density analysis, dendrites of pyramidal neurons in cortical layer II/III at P60 were examined. The number of spines on each dendrite at between 30 μm and 80 μm from the soma was counted, and the spine density was represented as the number of spines per dendrite length of 10 μm. All images were acquired using a FV1000 (Olympus) or LSM880 (Carl Zeiss, Göttingen, Germany) confocal laser scanning microscope. Image processing was performed with Fiji (http://fiji.sc) and Photoshop (Adobe Systems, San Jose, CA).

### Statistical analyses

Statistical significance was determined using one-way ANOVA followed by a Dunnett’s post hoc test for multiple comparisons using Prism 8 (GraphPad Software, San Diego, CA).

## Results

**Fig. 1:**
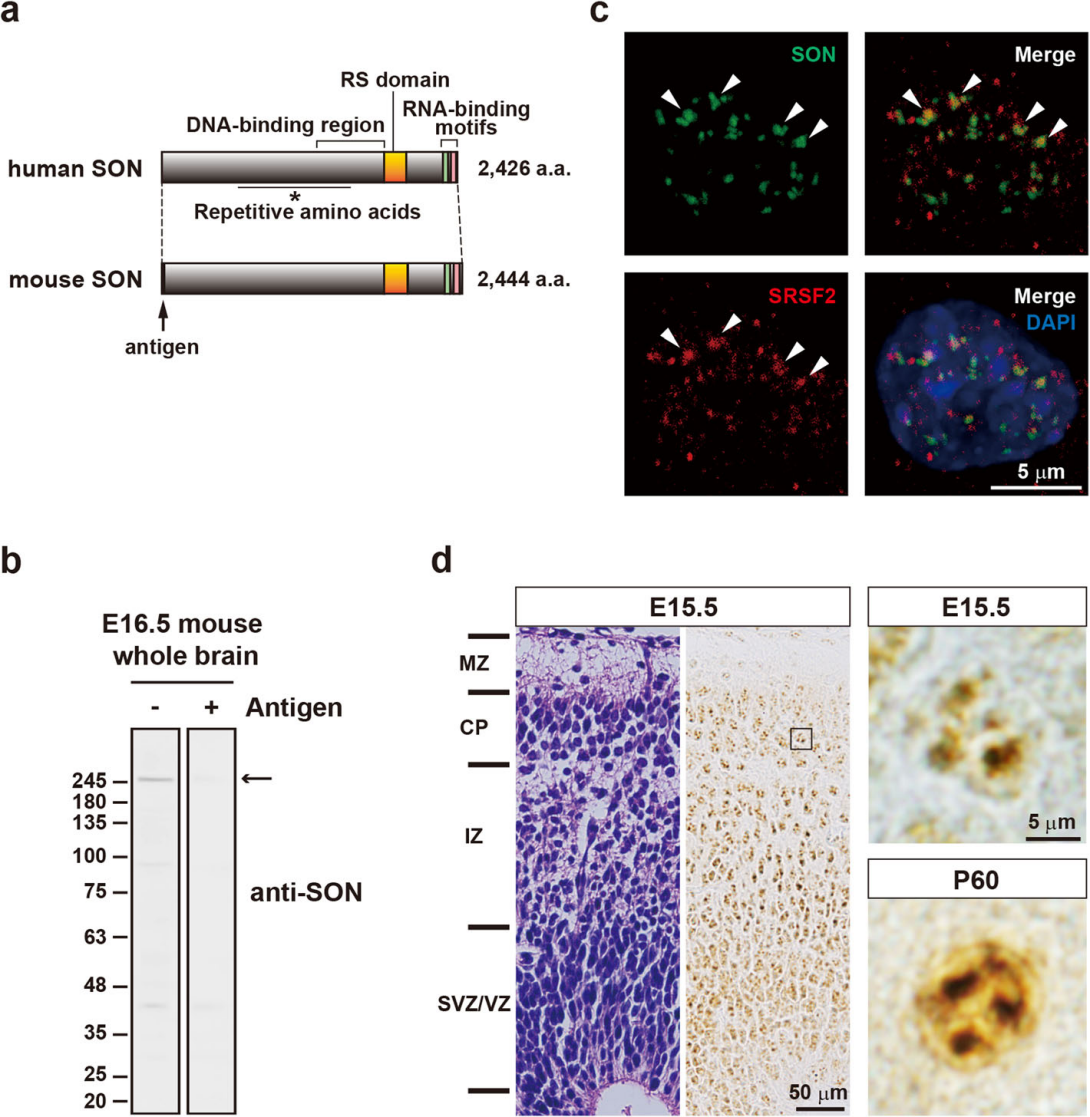
SON distribution in neural cells and in the developing mouse brain cortex. (**a**) A schematic representation of the structure of full-length human and mouse SON. The arrow indicates the portion used as an antigen for antibody production. (**b**) Characterization of the anti-SON antibody. E16.5 mouse brain lysates were used for Western blotting. The antibody detected multiple bands. The major band, the 260-kDa band indicated by the arrow, was almost completely absent after the antibody was absorbed with glutathione s-transferase-fusion antigen peptide (right lane). (**c**) The localization of SON in the nucleus of Neuro-2a cells. Cells were stained with an anti-SON (green) or anti-SRSF2 (red) antibody. The nuclei were stained with DAPI (blue). The arrowheads indicate the colocalization of SON and SRSF2. (**d**) Immunohistochemical distribution of SON in the developing mouse brain and its subcellular localization in mature neurons. The left panel shows a hematoxylin and eosin (HE)-stained section of the E15.5 mouse cerebral cortex. The layered structure of the developing cortex is shown on the left. MZ: marginal zone; CP: cortical plate; IZ: intermediate zone; SVZ: subventricular zone; VZ: ventricular zone. The middle panel shows the distribution of SON in an adjacent section to the HE-stained section. At E15.5, most neuronal progenitors and neurons expressed SON. The right upper panel shows a higher magnification view of the boxed area in the middle panel. SON was localized to the nucleus and was present as speckles, as in cultured cells. A similar subcellular distribution of SON was observed in mature neurons at P60 (the left lower panel)

The human *SON* and mouse *Son* genes encode 2426-amino-acid and 2444-amino-acid proteins, respectively, and share 84.2% homology [13] (Fig. 1a). We raised an antibody against mouse SON and found that it recognized a major band of approximately 260 kDa, which corresponds to the predicted size, and a few lower molecular weight bands upon Western blot analysis of mouse embryonic brain lysates (Fig. 1b). These bands were almost completely absent after the antibody was preabsorbed with the antigen peptide, confirming its specificity (Fig. 1b, right panel). In immunocytochemistry, the antibody recognized nuclear speckles in Neuro-2a cells, and the signals were partially colocalized with SRSF2, a splicing factor that, with SON, forms the core of speckles [17], further confirming the specificity of the antibody (Fig. 1c). The antibody worked well for immunohistochemistry as well, and we found that the most cells in the developing mouse brain at E15.5 expressed SON and that SON signals were especially conspicuous as nuclear speckles in presumptive migrating neuronal progenitors in the intermediate zone (IZ) and neurons in the cortical plate (Fig. 1d). In addition, we confirmed that the SON expression was maintained in mature neurons at P60 (Fig. 1d).

**Fig. 2:**
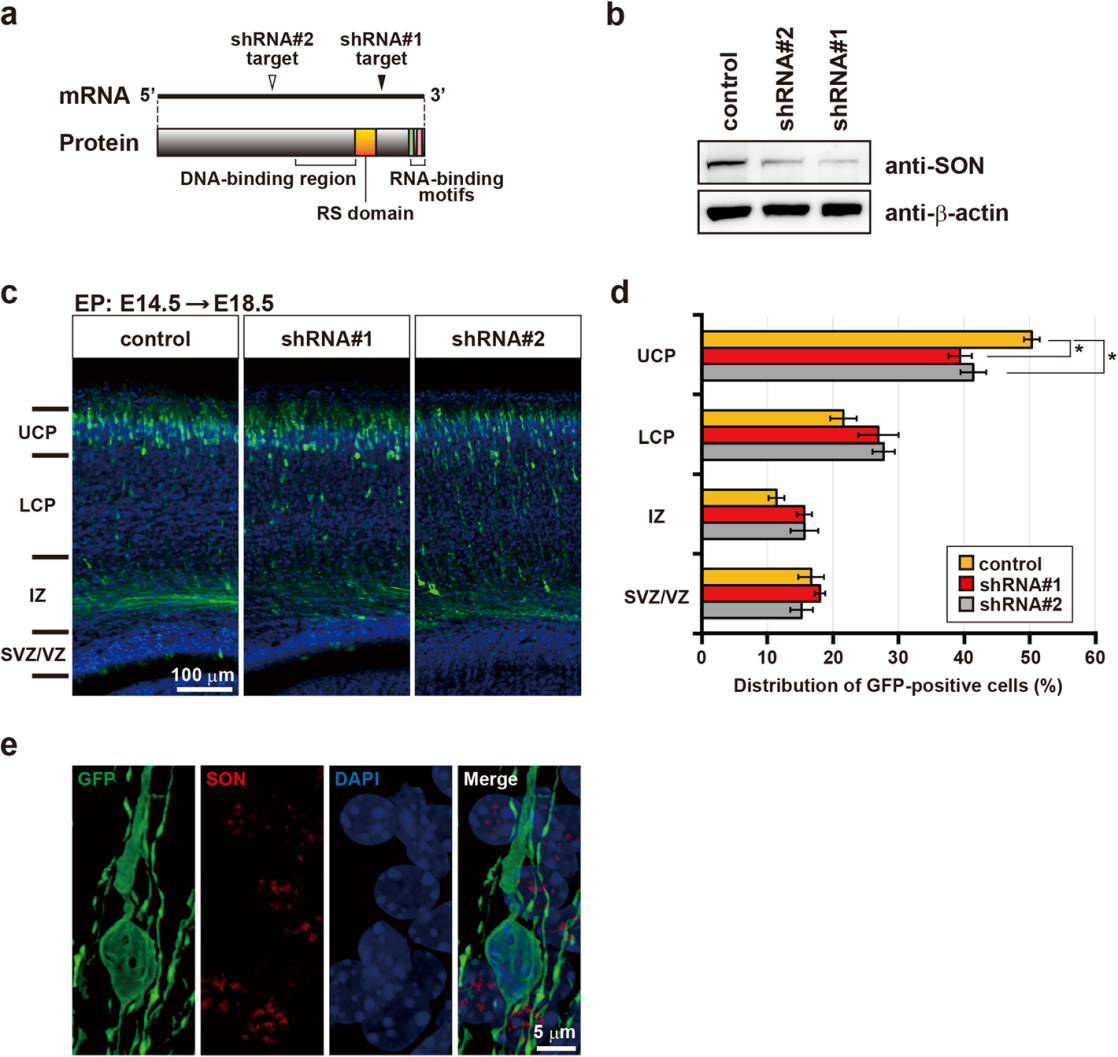
SON is necessary for normal neuronal migration. **a** A schematic representation of the shRNA target sites. The closed and open arrowheads indicate the target positions of shRNA#1 and shRNA#2, respectively, on *SON* mRNA. **b** Characterization of *Son* shRNA-expressing vectors. Neuro-2a cells were transfected with parental pLLC vector (control) or vectors engineered to express *Son* shRNAs (shRNA#1 or shRNA#2). Cell lysates were subjected to Western blotting with an anti-SON antibody to assess the effect of knockdown. β-actin was used as a loading control and was used to normalize quantified values of SON signals. **c** Representative images showing the distribution of GFP-positive cells transfected with either empty vectors (control) or a vector expressing shRNA#1 or shRNA#2 in the developing mouse cerebral cortex. The layered structure is shown on the left. UCP: upper cortical plate; LCP: lower cortical plate; other abbreviations as described in Fig. 1d. The cortical plate is divided into the UCP and LCP according to cell density [18]. Transfection was performed by IUE at E14.5. Coronal brain sections were prepared at E18.5 and stained for GFP (green) and DAPI (blue). **d** The quantification of GFP-positive cells in each layer of the developing cortex. Each layer is described as in (**c**). Total numbers of GFP-positive cells studied in each brain ranged 306–567. Error bars represent standard error of the mean (SEM). *n* = 4; *p < 0.05 by one-way ANOVA followed by Dunnett’s test. The original data are available in Additional file 1 [Table S1]. **e** The confirmation of *Son* knockdown in shRNA-introduced neurons. Coronal sections prepared as described in (**c**) were stained for GFP (green), SON (red), and DAPI (blue)

To reveal the functional significance of SON in neural progenitors, we then applied IUE to knockdown *Son* specifically in these cells and examined the effects on migration. We generated two shRNA expression constructs targeting independent sites of *Son* mRNA (Fig. 2a) and confirmed that both shRNAs reduced SON expression levels in Neuro-2a cells to 17.8% (shRNA#1) and 32.6% (shRNA#2) of that in the control (Fig. 2b). IUE was performed at E14.5 to deliver shRNA#1 or shRNA#2 to neural progenitors, and the distribution of SON knockdown cells in the developing cortex at E18.5 was examined. As shown in Fig. 2c, GFP-positive *Son* knockdown neurons (shRNA#1 and shRNA#2) in the upper cortical plate (UCP) were sparse compared with GFP-positive neurons without *Son* knockdown (control). The quantification of GFP-positive cells in each cortical layer revealed that fewer *Son* knockdown neurons than control neurons were distributed in the UCP (Fig. 2d). Although slightly more *Son* knockdown cells than control cells were distributed in the lower cortical plate (LCP) and IZ, the differences were not statistically significant. In addition, we examined SON expression levels in GFP-positive shRNA-introduced cells in electroporated samples, and confirmed that the SON signals in GFP-positive cells was hardly detectable, while that in GFP-negative cells was clearly observed as nuclear speckles (Fig. 2e). These results indicate that canonical *Son* expression in neural progenitors is indispensable for normal neuronal migration.

**Fig. 3:**
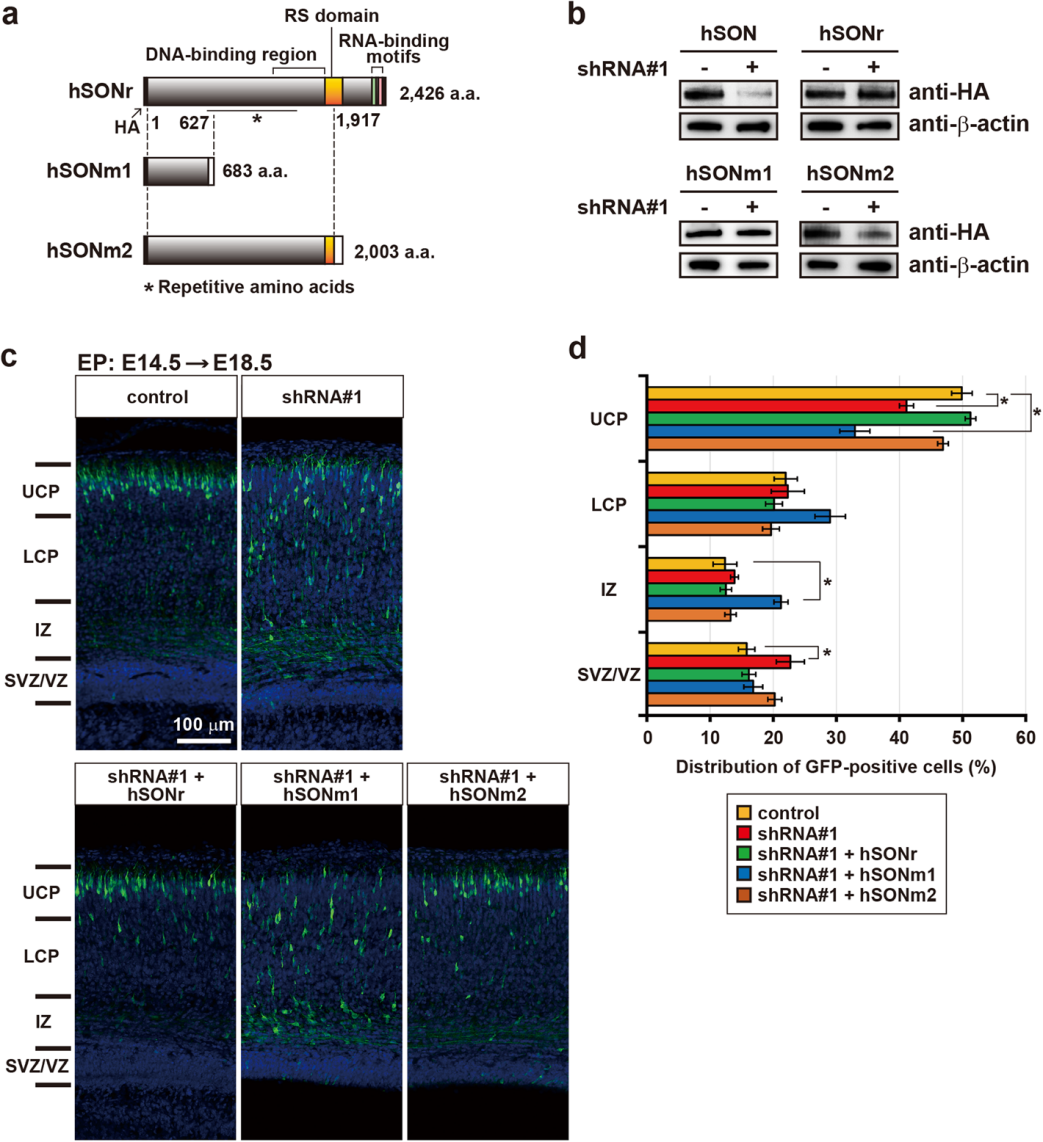
Wild-type human SON and a disease-associated mutant SON rescue migration defects induced by *Son* knockdown. **a** Schematic representations of the structures of shRNA-resistant human SON (hSONr) and disease-associated mutant SON proteins (hSONm1 and hSONm2). (**b**) The confirmation of shRNA resistance of various forms of human SON. HEK293 cell lysates expressing various forms of hSON with or without shRNA#1, as described above, were subjected to Western blotting with an anti-HA antibody. β-actin was used as a loading control. **c** Representative images showing the distribution of GFP-positive cells in the rescue experiments. The layered structure is shown as in Fig. 2c. Vectors expressing shRNA with or without those expressing various forms of hSON described in the boxes above each image were transfected into neural progenitors at E14.5. Parental pLLC vectors expressing GFP were used as a control. Coronal sections were prepared at E18.5 and stained as in Fig. 2c. **d** The quantification of GFP-positive cells in each layer of the developing cortex. Each layer is described as in (**c**). Total numbers of GFP-positive cells studied in each brain ranged 161–757. Error bars represent the SEM. *n* ≥ 3; *p < 0.05 by one-way ANOVA followed by Dunnett’s test. The original data are available in Additional file 1 [Table S2]

Next, we performed rescue experiments by overexpressing shRNA-resistant human SON (hSONr) in knockdown cells. In addition, we examined constructs expressing two forms of disease-associated mutant SON. hSONm1 is a truncated mutant without most of the known functional domains, while hSONm2 lacks RNA-binding motifs and the C-terminal half of the RS domain (Fig. 3a). The former is derived from a *SON* mutation reported by Kim et al. [1], and the latter is from the most prevalent mutation found in ZTTK syndrome [1, 3, 4]. The effective production of hSONr, hSONm1, and hSONm2 in HEK293 cells in the presence of shRNA#1 was confirmed (Fig. 3b). Then, the vectors expressing wild-type or mutant SON were introduced along with shRNA vectors into neural progenitors at E14.5, and the distribution of GFP-positive cells was examined. As shown in Fig. 3c and d, migration defects induced by shRNA#1 were rescued by the overexpression of hSONr (shRNA#1 + hSONr), confirming that the defects were caused by SON insufficiency in neural progenitors. Intriguingly, the overexpression of hSONm2 (shRNA#1 + hSONm2), but not that of hSONm1 (shRNA#1 + hSONm1), rescued the defects as well, indicating that hSONm2, like hSONr, exerts sufficient functions for neuronal migration, while hSONm1 does not.

**Fig. 4:**
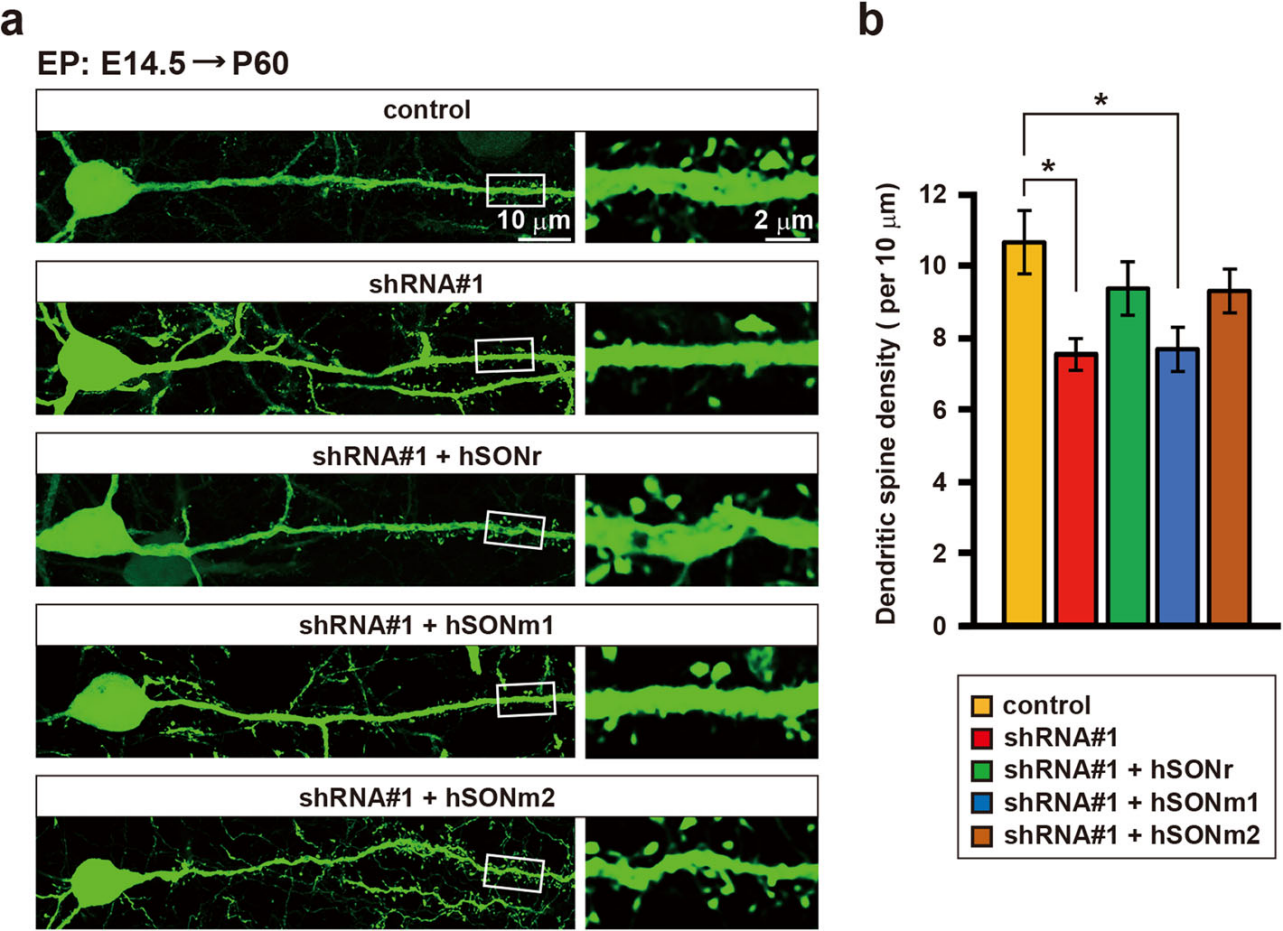
*Son* knockdown causes a reduction in spine density on cortical pyramidal neurons. **a** Representative images showing pyramidal neurons in layers II/III at P60. Vectors expressing shRNA#1 with or without those expressing wild-type (hSONr) or mutant hSON (hSONm1 or hSONm2), as described in the boxes above each image, were transfected into neural progenitors at E14.5. Parental pLLC vectors were used as controls. Coronal cortical sections were prepared at P60 and stained for GFP (green). The right panels are high magnification views of the boxed areas in the left panels. **b** Quantification of the spine density. The number of spines on each dendrite at between 30 μm and 80 μm from the soma was counted. The spine density is represented as the number of spines per dendrite length of 10 μm. Error bars represent the SEM. One-way ANOVA followed by Dunnett’s test was used for each statistical analysis. *p < 0.05, *n* ≥ 17 neurons. The original data are available in Additional file 1 [Table S3]

Since ID is not always accompanied by cerebral cortical malformation due to migration abnormalities, we reasoned that *SON* haploinsufficiency affects other factors essential for intellectual abilities. Therefore, we examined the dendritic spine density on *Son* knockdown neurons. The density of dendritic spines on *Son* knockdown cortical neurons at P60 (7.6 ± 0.5 per 10 μm) was decreased by approximately 30% compared to that on control neurons (10.6 ± 0.9 per 10 μm) (Fig. 4a, b). The forced expression of hSONr resulted in a dendritic spine density nearly equal to that on control neurons, confirming that SON insufficiency resulted in decreasing in the numbers of dendritic spines (Fig. 4a, b). These data indicate that SON is necessary for normal spine formation during neural development and that *SON* haploinsufficiency may cause dendritic spine abnormalities. Again, the overexpression of hSONm2, but not that of hSONm1, rescued the abnormalities as well, indicating that hSONm2 retains functions necessary for spine formation, while hSONm1 does not (Fig. 4a, b).

## Discussion

In this report, we clarified that the canonical expression of *Son* is necessary for normal neuronal migration and dendritic spine formation in the developing mouse cerebral cortex. In addition, a truncated form of SON encoded by the most prevalent mutant *SON* identified in ZTTK syndrome patients can ameliorate the neuronal abnormalities induced by *Son* knockdown. These findings provided some hints to understand the pathophysiological mechanisms underlying the neural symptoms of ZTTK syndrome.

Among the 31 de novo mutations in the *SON* gene reported to be associated with ZTTK syndrome so far, twenty-eight encode truncated SON proteins due to either frameshift or nonsense substitutions that generate premature termination codons (Table 1). These truncated SON proteins, if produced, vary in length, with their C-termini distributed widely over the normal full-length SON protein and function differently from one another. However, mRNAs bearing a premature termination codon are often targeted by NMD, and are degraded [19]. Kim et al. revealed that some mutant *SON* mRNAs and proteins are indeed highly downregulated in the peripheral blood mononuclear cells of patients [1], suggesting that ZTTK syndrome is caused by *SON* haploinsufficiency; truncated proteins encoded by mutant *SON* are not involved in pathogenesis of ZTTK syndrome. The pathophysiological consequence of *SON* insufficiency in brain development is therefore of great interest to understand the neural symptoms of ZTTK syndrome. In this respect, our finding that the number of neurons that migrated into the UCP decreased by approximately 20% due to *Son* knockdown provides a concrete evidence that SON insufficiency in neural progenitors results in migration defects, which seems to be the pathological basis of brain malformation in ZTTK syndrome. More importantly, we found reduced spine density on *Son* knockdown neurons. Dendritic spine dysgenesis, such as a reduction in the number of spines and morphological abnormalities of spines, in cortical neurons was originally reported as a common pathological feature found in mentally retarded children with normal karyotypes [20]. The similar spine abnormalities of cortical neurons were identified also in genetic disorders associated with mental retardation, a former name for ID (reviewed in [21]). Finally, the search for genetic causes of ID has identified numerous mutated genes that may be involved in synapse formation and the regulation of dendritic spine morphology [22]. To our knowledge, postmortem reports of ZTTK syndrome patients are unavailable. Therefore, reduced spine density on *Son* knockdown neurons is an important finding and suggests that spine defects are the pathological basis of ID in ZTTK syndrome.

**Table 1:** Summary of mutation types identified in *SON* in association with ZTTK syndrome

Mutation type	cDNA	Protein (predicted)	No. of case	Reference
Frame-shift deletion (20 cases)	c.268del	p.Ser90Valfs*59	1	[1]
	c.1881_1882del^a^	p.Val629Alafs*56	1	[1]
	c.2365del	p.Ser789Alafs*8	1	[1]
	c.3597_3598del	p.Pro1200Argfs*17	1	[1]
	c.3852_3856del	p.Met1284Ilefs*2	2	[1, 4]
	c.4055del	p.Pro1352Glnfs*14	1	[1]
	c.4358_4359del	p.Thr1453Serfs*11	1	[1]
	c.4640del	p.His1547Leufs*76	1	[1]
	c.4678del	p.Glu1560Lysfs*63	1	[1]
	c.5549_5550del	p.Arg1850Ilefs*3	1	[1]
	c.5753_5756del^a^	p.Val1918Glufs*87	7	[1, 3, 4]
	c.6087del	p.Ser2029Argfs*22	1	[1]
	c.6233del	p.Pro2078Hisfs*4	1	[4]
Frame-shift insertion (2 cases)	c.[4999_5013del; 5031_5032insAA]^b^	p.[Asp1667_Asn1671del; Asp1678Lysfs*9]	1	[1]
	c.6002_6003insCC	p.Arg2002Glnfs*5	1	[1]
Frame-shift duplication (2 cases)	c.3073dup	p.Met1025Asnfs*6	1	[4]
	c.4549dup	p.Glu1517Glyfs*6	1	[1]
Nonsense substitution (4 cases)	c.286C > T	p.Gln96*	1	[4]
	c.394C > T	p.Gln132*	1	[5]
	c.3334C > T	p.Arg1112*	2	[1, 2]
In-frame deletion	c.4151_4174del24	p.Leu1384_Val1391del	1	[1]
Missense substitution	c.[4909A > T; 5528C > A]^c^	p.[Thr1637Ser; Ser1848Thr]	1	[4]
Whole gene deletion	–	–	1	[1]

^a^ These two types of mutation were examined in this study. ^b^ An in-frame deletion and a frame-shift insertion were identified in one allele; the latter was regarded as pathogenic. ^c^ Two substitutions were identified in one allele

Rescue experiments that involved the introduction of two forms of disease-associated mutant SON proteins confirmed that the truncated SON proteins encoded by mutant *SON* genes differ in their residual functions, even though both mutations cause ZTTK syndrome. As shown in Fig. 3a, hSONm1 is a severely truncated SON with 683 amino acids and hSONm2 is a mildly truncated SON with 2003 amino acids. Previous studies have revealed that the RS domain and the G-patch that constitutes RNA-binding motifs are the core motifs necessary for SON mediated RNA splicing [7] and a middle portion partially overlapping with a unique amino acid repeats is the DNA-binding region that bind to human genomic and viral DNAs [9, 23]. Based on the domain structure, hSONm2 lacking an RNA-binding motif and part of the RS domain is expected to be functional in part, as the DNA-binding domain remains intact. In contrast, hSONm1 lacking most of the identified domains were supposed to be hardly functioning. Indeed, the overexpression of hSONm2, like wild-type SON, successfully rescued the neuronal abnormalities, while the overexpression of hSONm1, failed to do so in our rescue experiments. These data indicate that the hSONm1-coding mutation is a loss-of-function mutation, but that the hSONm2-coding mutation retains function comparable, at least in the neural development, to that of wild-type SON. As mentioned above, it is suggested that ZTTK syndrome is caused by *SON* haploinsufficiency; potentially functional proteins encoded by mutant *SONs* are not involved in pathogenesis of ZTTK syndrome due to NMD [1]. It is, therefore, not surprising that hSONm2 encoded by the most prevalent mutant *SON* associated with ZTTK syndrome retained considerable function in neural development. Rather, the finding may further support noninvolvement of truncated proteins encoded by disease-associated mutant SONs in pathogenesis of ZTTK syndrome.

The molecular mechanisms underlying the neural abnormalities caused by *SON* mutations remain unclear because the roles of the multifunctional nuclear protein SON are diverse and not fully understood. The finding that hSONm2, which lacks an RNA-binding motif and part of the RS domain, behaved like wild-type SON in the rescue experiments may provide hints for these mechanisms. A simple explanation for this result is that the loss of the RNA splicing function of SON does not play a significant role in the observed neuronal abnormalities. Instead, other functions, such as transcriptional regulation, are more relevant to neural pathology since SON interacts with more than a thousand of genes via its DNA-binding region and is involved in the transcriptional repression of many target genes [9]. This is supported by the fact that rare non-truncating mutations, i.e., missense mutations [4] and an in-frame deletion [1], identified in ZTTK syndrome patients, are located exclusively in and around the genomic region that encodes the DNA-binding region (Table 1). However, there is a possibility that hSONm2 influences SON-mediated RNA splicing because of its structural similarity to SON E, a physiological isoform of SON. This truncated isoform, which lacks an RNA-binding motif, has been reported to enhance full-length SON-mediated RNA splicing [9]. Therefore, it is possible that hSONm2 together with residual mouse full-length SON rescues RNA splicing deficits caused by SON insufficiency. Many more investigations are necessary to understand the detailed molecular mechanisms underlying the neural pathology of ZTTK syndrome, and accumulation of clinical and genetic information of ZTTK syndrome is also important.

In conclusion, this study revealed clearly that *Son* insufficiency results in neuronal migration defects and dendritic spine abnormalities in the mouse brain. Since information about the neuropathology of ZTTK syndrome is extremely limited, these findings provide important neuropathological basis possibly responsible for the neural symptoms, i.e., brain malformation and ID, of ZTTK syndrome. In addition, rescue experiments provided further evidence supporting that putative neural abnormalities in ZTTK syndrome are caused by *SON* haploinsufficiency regardless of the residual functions of mutant *SON* genes.

## Supplementary information

**Additional file 1: Table S1.** Numbers of GFP-positive cells in each layer of the developing cortex (%) **Table S2.** Numbers of GFP-positive cells in each layer of the developing cortex (%) **Table S3.** Dendritic spine density values (per 10 μm of dendrite) of each neuron

## Data Availability

All original data to generate Figs. 2, 3 and 4d and b are included in this published article [Table S1, S2, and S3 in Additional file 1 “Supplemental Tables”]. Materials are available on request.

## Abbreviations

GFP: Green fluorescent protein
HA: Hemagglutinin
HE: Hematoxylin and eosin
HRP: Horseradish peroxidase
ID: Intellectual disability
IUE: In utero electroporation
IZ: Intermediate zone
LCP: Lower cortical plate
MZ: Marginal zone
NMD: Nonsense-mediated mRNA decay
PBS: Phosphate-buffered saline
SEM: Standard error of the mean
shRNA: Small hairpin RNA
SVZ: Subventricular zone
UCP: Upper cortical plate
VZ: Ventricular zone
ZTTK: Zhu-Tokita-Takenouchi-Kim
